# Associations Between High-Density Lipoprotein Cholesterol Efflux and Brain Grey Matter Volume

**DOI:** 10.3390/jcm13206218

**Published:** 2024-10-18

**Authors:** John M. Giacona, Jijia Wang, Rong Zhang, Brendan J. Kelley, Ihab Hajjar, Binu P. Thomas, Fang F. Yu, James A. de Lemos, Anand Rohatgi, Wanpen Vongpatanasin

**Affiliations:** 1 Hypertension Section, Department of Internal Medicine, University of Texas Southwestern Medical Center, Dallas, TX 75235, USA; 2 Department of Applied Clinical Research, University of Texas Southwestern Medical Center, Dallas, TX 75235, USA; jijia.wang@utsouthwestern.edu; 3 Cardiology Division, University of Texas Southwestern Medical Center, Dallas, TX 75235, USA; james.delemos@utsouthwestern.edu (J.A.d.L.); anand.rohatgi@utsouthwestern.edu (A.R.); 4 Institute for Exercise and Environmental Medicine, Texas Health Presbyterian Hospital, Dallas, TX 75235, USA; rongzhang@texashealth.org; 5 Department of Neurology, University of Texas Southwestern Medical Center, Dallas, TX 75235, USA; brendan.kelley@utsouthwestern.edu; 6 Department of Neurology and Neurotherapeutics, University of Texas Southwestern Medical Center, Dallas, TX 75235, USA; ihab.hajjar@utsouthwestern.edu; 7 Advanced Imaging Research Center, University of Texas Southwestern Medical Center, Dallas, TX 75235, USA; binu.thomas@utsouthwestern.edu; 8 Department of Radiology, University of Texas Southwestern Medical Center, Dallas, TX 75235, USA; frankf.yu@utsouthwestern.edu

**Keywords:** high-density lipoprotein cholesterol, brain grey matter, neurodegeneration, neurocognitive function

## Abstract

**Objective:** High-density lipoprotein cholesterol efflux function may prevent brain amyloid beta deposition and neurodegeneration. However, the relevance of this finding has not been established in the diverse middle-aged population. **Methods:** We examined 1826 adults (47% Black adults) who participated in the Dallas Heart Study to determine associations between high-density lipoprotein (HDL) measures and brain structure and function. White matter hyperintensities (WMH) and whole-brain grey matter volume (GMV) were measured using brain MRI, and the Montreal Cognitive Assessment (MoCA) was used to measure neurocognitive function. HDL cholesterol efflux capacity (HDL-CEC) was assessed using fluorescence-labeled cholesterol efflux from J774 macrophages, and HDL particle size measures were assessed using nuclear magnetic resonance (NMR) spectroscopy (LipoScience). Multivariable linear regressions were performed to elucidate associations between HDL-CEC and brain and cognitive phenotypes after adjustment for traditional risk factors such as age, smoking status, time spent in daily physical activity, and education level. **Results:** Higher HDL-CEC and small HDL particle (HDL-P) concentration were positively associated with higher GMV normalized to total cranial volume (TCV) (GMV/TCV) after adjustment for relevant risk factors (β = 0.078 [95% CI: 0.029, 0.126], *p =* 0.002, and β = 0.063 [95% CI: 0.014, 0.111], *p =* 0.012, respectively). Conversely, there were no associations between HDL measures and WMH or MoCA (all *p* > 0.05). Associations of HDL-CEC and small HDL-P with GMV/TCV were not modified by ApoE-ε4 status or race/ethnicity. **Interpretation:** Higher HDL cholesterol efflux and higher plasma concentration of small HDL-P were associated with higher GMV/TCV. Additional studies are needed to explore the potential neuroprotective functions of HDL.

## 1. Introduction

Several epidemiological studies have demonstrated associations of plasma lipids during early and midlife with the development of cognitive decline and dementia in late life [1,2,3,4]. Notably, low levels of high-density lipoprotein cholesterol (HDL-C) have been associated with impaired cognitive function [5,6,7] and the presence of amyloid beta (β) deposits [8] and parahippocampal atrophy [9] in the brain that are associated with dementia [10]. In contrast, some studies have observed no association between HDL-C and impaired cognitive function [11,12,13]. Importantly, HDL particles exert antioxidant and anti-inflammatory effects and play an important role in the removal of excess cholesterol from vascular cells through reverse cholesterol transport [14]. Greater HDL cholesterol efflux capacity (HDL-CEC), a measure of HDL reverse cholesterol transport functionality, associates linearly with reduced cardiovascular disease risk independently from HDL-C and of Apolipoprotein A-I (ApoA-I), the main constituent protein of HDL particles [15,16]. Excess levels of cholesterol in the central nervous system promote neurotoxicity and the development of Alzheimer’s disease (AD) by enhancing amyloid β deposition [17], and this is reflected in the reduced HDL-CEC levels found in the cerebrospinal fluid of individuals with mild cognitive impairment (MCI) and AD [18,19,20].

However, the association between plasma HDL-CEC or HDL composition and brain structure has not been investigated. Although one prior study investigated the association between plasma HDL-CEC and cognitive function, it involved a small sample of participants that included 50 cognitively normal adults [18]. The small sample size limited the ability to account for the confounding influence of relevant variables. To address this, we used a large, multiethnic cohort of middle-aged adults from the Dallas Heart Study to evaluate the associations between HDL measures and brain structure and function.

## 2. Materials and Methods

All investigations were approved by the Institutional Review Board at the University of Texas Southwestern Medical Center and conducted in participants of the Dallas Heart Study after informed consent was obtained. In summary, the Dallas Heart Study (DHS) is a multiethnic, population-based, probability sample of Dallas County, Texas, U.S.A. The details of the Dallas Heart Study (DHS) participant selection criteria, overall study design, and detailed methods, have been previously described [21]. As DHS was a population-based cohort study of Dallas County adults, there were no exclusion criteria, while all adults aged 35–70 years were eligible for enrollment. Participants of the DHS phase-1 (DHS-1) that occurred between 2000 and 2002, and the longitudinal follow-up DHS phase-2 (DHS-2) that occurred between 2007 and 2009, were eligible to be included in the present study (NCT00344903). All participants had fasting lipoprotein measures assessed by nuclear magnetic resonance (NMR). High-density lipoprotein (HDL) cholesterol efflux capacity (CEC) was assessed using J774 macrophages, fluorescence-labeled cholesterol, and Apolipoprotein B-depleted plasma during both phases (DHS-1 and DHS-2). Participants underwent cognitive testing (Montreal Cognitive Assessment; MoCA) and brain magnetic resonance imaging (MRI) during a single visit of DHS-2. Race was self-reported by all participants of this study, and race categories were defined based on the United States Office of Management and Budget’s Revisions to the Standards for the Classification of Federal Data on Race and Ethnicity.

### 2.1. Lipoprotein Characterization and HDL-Cholesterol Efflux Capacity (HDL-CEC)

Fasting blood samples collected by venipuncture was placed in ethylenediaminetetraacetic acid tubes, centrifuged, and the plasma was removed and stored at −70 °C [22]. Plasma lipids, including HDL cholesterol, were assessed as previously described [22]. HDL particle concentration and size were likewise described previously [15]. HDL cholesterol efflux capacity was assessed by measuring fluorescence-labeled cholesterol efflux from J774 macrophages to apolipoprotein B-depleted plasma, as previously described [23].

### 2.2. Brain Magnetic Resonance Imaging (Brain MRI)

The DHS brain MRI protocol was performed on a 3.0 Tesla MR imaging unit (Achieva; Philips Medical Systems, Memphis, TN, USA) with acquisition of two-dimensional, fluid attenuated inversion recovery (FLAIR) images obtained with an echo time of 130 ms, a repetition time of 11,000 ms, and an inversion time of 2800 ms; a sensitivity encoding factor of 2; a echo train length of 44; a field of view of 250 × 250 mm; and 4 mm sections spaced at 5 mm centers. Three-dimensional magnetization prepared rapid acquisition with gradient echo (MPRAGE) images that were obtained with an echo time of 5.8 and a repetition time of 9.6 ms; a sensitivity encoding factor of 2; a flip angle of 12°; a field of view of 260 × 260 mm; and 2 mm sections spaced at 1 mm centers. Further details are as previously described [24]. The morphologic variables selected for the present analysis were total cranial volume (TCV), white matter hyperintensities (WMH), and whole-brain grey matter volume (GMV). Structural brain imaging measures used for this analysis were acquired through regional quantification of brain volumes using the freely available Functional MRI of the Brain Software Library (FSL v6.0) (http://fsl.fmrib.ox.ac.uk/fsl/fslwiki/, accessed on 2 September 2024) [24,25,26].

### 2.3. The Montreal Cognitive Assessment (MoCA)

Total MoCA score is a 30-point screening tool to assess cognitive function [27], which includes assessment of attention, verbal memory, orientation, language, visuospatial, and executive function [28]. The MoCA was administered by trained research personnel in the DHS-2 and double-checked for accuracy, as previously described [27].

### 2.4. Physical Activity Assessment

A wrist-based accelerometer (Actical, Philips Respironics, Bend, OR, USA) was used to measure time spent in moderate-to-vigorous physical activity (MVPA) for participants enrolled in the Dallas Heart Study between 2008 and 2009, as previously described [21].

### 2.5. Apolipoprotein E (ApoE) Genotyping

TaqMan single-nucleotide polymorphism genotyping assays (Applied Biosystems, Foster City, CA, USA) were used to genotype c.388T> C (rs429358) and c.526C > T (rs7412), that define three haplotypes ε2(388 T–526 T), ε3(388 T-526C), and ɛ4(388C-526C) from genomic DNA that was extracted from circulating leukocytes. Positive carrier status for ApoE-ε4 allele was defined as ε2/ε4, ε3/ε4, or ε4/ε4 and negative as ε2/ε2, ε2/ε3, or ε3/ε3.

### 2.6. Statistical Analysis

Statistical analyses were performed using SAS version 9.4 (Carey, NC, USA). Participant characteristics are reported as the mean ± SD (standard deviation), or mean with 95% SD, for continuous data. Frequency with percentage (%) are reported for categorical data. The Mann–Whitney U test and the two-sample t-test were used to compare continuous baseline measurements while the Chi-squared test was used to compare categorical baseline measurements. Multivariable linear regression analyses were conducted to assess associations between lipid exposures and brain and cognitive outcomes. Standardized beta estimates with 95% confidence intervals (CI) were reported. The standardized beta predicted the change in the dependent variable for 1 SD of change in the independent variable.

Multivariable linear regression analyses were performed to assess the associations between HDL and lipoprotein measures averaged from both DHS phases and total MoCA score, WMH/TCV, and GMV/TCV from DHS-2. Models were performed with a single HDL parameter and were adjusted for the following covariates: age, sex, race/ethnicity, smoking status, education level, and MVPA from DHS-2; and mean systolic blood pressure (SBP), fasting plasma glucose, body mass index (BMI), and estimated glomerular filtration rate averaged from DHS-1 and DHS-2. The use of mean exposure variables as well as the mean levels of covariates during the follow-up between DHS-1 and DHS-2 allow for assessment of the cumulative impact of cardiovascular risk factors on the brain structure and function outcomes, which was used previously in longitudinal studies [29,30]. Education levels were categorized into four groups: (1) less than high school education, (2) high school education, (3) college education, and (4) above college degree. All modeling assumptions were verified utilizing diagnostic tools (residual versus predicted value plot, Cook’s distance, and Q–Q plot). Similar models were performed to test interactions between race/ethnicity and risk factors by including multiplicative interaction terms. Since GMV and WMH data were skewed, analyses were performed after log transformation. Restricted cubic splines of association between cardiac and brain variables were constructed. Statistical significance was set a priori as *p*-value < 0.05.

## 3. Results

### 3.1. Participant Characteristics

**Figure 1:**
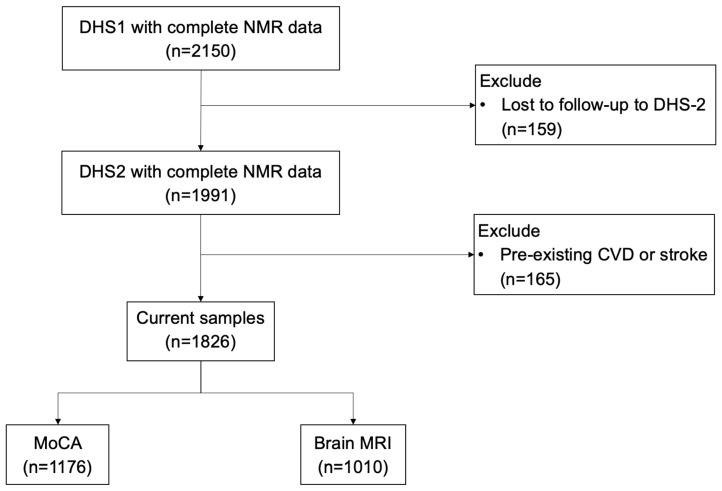
Study Flow Chart (CVD: cardiovascular disease; DHS: Dallas Heart Study; MRI: magnetic resonance imaging; NMR: nuclear magnetic resonance; MoCA: Montreal Cognitive Assessment).

As shown in Figure 1, there were 2150 participants in DHS-1 that had complete HDL particle measurement by NMR, of whom 159 were lost to follow-up and did not participate in DHS-2. Of the participants in DHS-1, there were 1991 who had participated in both phases (DHS-1 and DHS-2). Among those, 165 were removed due to prior myocardial infarction, cardiac arrest, or stroke, leaving 1826 adults included for analyses. Among these remaining participants, 1010 underwent brain MRI and 1176 underwent the Montreal Cognitive Assessment (MoCA).

The characteristics of the study participants are shown in Table 1. Overall, the mean age at the DHS-2 visit was 51.0 ± 9.7 years, with the cohort being 58% female, and 47% Black adults. Approximately 21% were current smokers and 32% were ApoE-ε4 carriers. Postsecondary education levels (i.e., college level and above) were reported by 63% of participants. The average lipid measures were as follows: mean HDL-C was 53.4 ± 12.8 mg/dL, total HDL particle (HDL-P) was 21.8 ± 3.2 μmol/L, small HDL-P was 15.0 ± 3.0 μmol/L, medium HDL-P was 4.7 ± 2.4 μmol/L, large HDL-P was 2.2 ± 1.4 μmol/L, and mean HDL-CEC was 0.93 ± 0.21 AU. The mean time spent engaging in MVPA was 39 (95% CI 37.2–40.7) minutes per day and the average systolic blood pressure was 128 ± 16 mmHg.

### 3.2. Association between HDL Measures and Brain Structure

**Figure 2:**
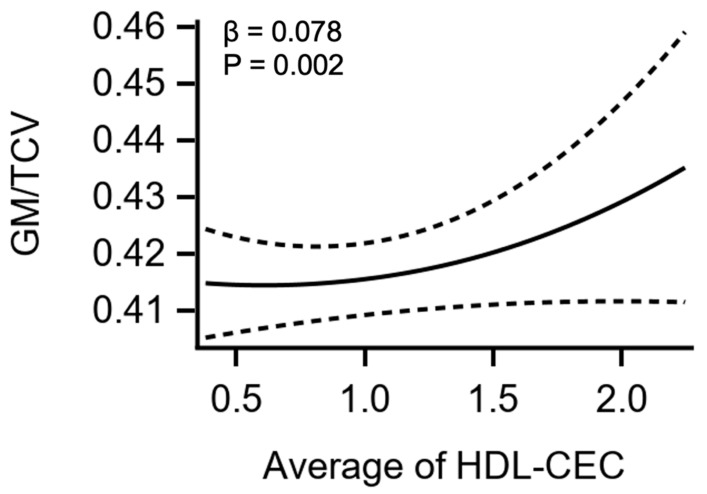
Cubic Spline of HDL-CEC with GM Volume. Restricted cubic spline and 95% confidence bands relating the average HDL-CEC with GMV in the whole cohort after adjustment for age, sex, race, MVPA, smoking status, education levels, and the average across DHS-1 and DHS-2 of BMI, systolic BP, plasma glucose, eGFR, and average HDL-C from both DHS phases. (BMI: body mass index; CEC: cholesterol efflux capacity; eGFR: estimated glomerular filtration rate; GMV: grey matter volume; HDL: high-density lipoprotein; MVPA: moderate-to-vigorous physical activity; BP: blood pressure; β: standardized regression coefficient.)

On multivariable regression, higher HDL cholesterol efflux capacity and higher circulating levels of small HDL-P were positively associated with greater GMV after normalization by TCV and adjustment for cardiovascular risk factors including age, smoking status, MVPA, and education (β = 0.078 [95% CI: 0.029, 0.126], *p* = 0.002, and β = 0.063 [95% CI: 0.014, 0.111], *p* = 0.012, respectively, Table 2, Figure 2). Conversely, there were no associations seen between total HDL-P, medium HDL-P, large HDL-P, HDL-C, or ApoAI with GMV after normalization by TCV and full adjustment in the whole cohort on multivariable regression analysis. The positive associations between HDL cholesterol efflux capacity and small HDL-P with GMV/TCV were not modified by ApoE-ε4 carrier status by race/ethnicity (all *p* > 0.05).

There were no associations between mean HDL-P, small HDL-P, medium HDL-P, large HDL-P, HDL-C, ApoAI, or HDL cholesterol efflux capacity with white matter hyperintensities normalized by total cranial volumes for the whole cohort (all *p* > 0.05, Table 2).

### 3.3. Association Between HDL Measures and Cognitive Function

No significant associations were observed between mean HDL-P, small HDL-P, medium HDL-P, large HDL-P, HDL-C, ApoAI, or HDL cholesterol efflux capacity with total MoCA score after adjustment for cardiovascular risk factors, education, and MVPA (all *p* > 0.05, Table 2).

## 4. Discussion

The main findings from our study are threefold. First, higher levels of HDL cholesterol efflux capacity, a measure of HDL reverse cholesterol transport functionality, was positively associated with greater grey matter volume after normalization to total cranial volume. Second, higher levels of circulating small HDL particles in the plasma were associated with greater grey matter volume. Third, the positive associations between HDL cholesterol efflux capacity and small HDL-P with grey matter volume were not modified by race/ethnicity or ApoE-ε4 carrier status.

Prior studies and meta-analyses have shown an inconsistent relationship between circulating plasma HDL-C and cognitive function or risk of dementia [5,6,7,13,31,32,33,34]. However, imaging studies have not been performed to investigate the impact of HDL composition or function on brain structure. Our study demonstrated that higher levels of HDL-CEC and plasma concentration of small HDL-P were positively associated with grey matter volume. This is significant, as previous studies have shown associations between GMV loss and the development of mild cognitive impairment (MCI) and the conversion of MCI to Alzheimer’s disease (AD) [35]. These findings may be explained by the observation that the majority of HDL (~95%) particles that crosses the blood–brain barrier are small HDL particles [36,37]. Once in cerebrospinal (CSF) fluid, small HDL particles play important roles in cholesterol biosynthesis, reducing inflammation, maintaining neural membrane integrity, and neuronal regeneration [38,39,40]. In the context of our findings, high HDL cholesterol efflux capacity and high levels of small HDL-P in the peripheral circulation likely reflect a metabolic milieu that is protective against neuronal loss and brain atrophy that are characteristic of neurodegenerative diseases.

Despite the significant association between HDL-CEC and small HDL with grey matter, no associations with MoCA score or white matter hyperintensities (WMH) were identified in our study. Previous studies have demonstrated plasma levels of small HDL are significantly lower in patients with AD when compared to healthy older adults, which aligns with our findings [36,41]. Moreover, the presence of high WMH volume generally represent cerebral small vessel disease, which is common among older adults with cardiovascular disease risk factors such as elevated blood pressure or stroke [42]. Thus, the inclusion of cognitively normal, younger participants (~50 years of age), and those free of clinical cardiovascular disease in our study likely contributed to the lack of association between our exposures and WMH. Reduced grey matter volume is a recognized risk marker for cognitive decline and the development of dementia in multiple populations [9,35,43]. Taken together, our findings support a beneficial role of HDL efflux capacity and circulating levels of small HDL in preserving grey matter volume and potential prevention of brain atrophy in healthy, diverse, middle-aged adults.

While some population studies have implicated plasma HDL-C in the pathogenesis of impaired cognitive function and dementia [44,45], the exact relationship remains controversial [11,12]. Our data did not identify associations between HDL-C with the MoCA, WMH, or GMV in the overall cohort. However, there were major differences in assessment of lipid measures, statistical design, and study population characteristics for the present study when compared to previous investigations [11,12,45]. Indeed, our study uniquely included serial measurements of lipoprotein variables to assess the cumulative impact of these risk factors in a middle-age and diverse cohort. Moreover, our study adjusted for several important confounders including race/ethnicity, smoking status, fasting plasma glucose, and time spent in daily physical activity, that were not consistently accounted for in prior studies, which may explain differences in results.

Our study had several strengths. The associations of the present study were observed in a large, population-based cohort of middle-aged adults, who were free from prior CVD or stroke, and appeared independent of traditional risk factors including age, smoking status, time spent in daily physical activity, and education level. Secondly, the focus of our study was placed on healthy, middle-aged individuals, where early-stage dementia risk may be present. Moreover, our study included serial measurements of HDL-CEC and lipoprotein concentrations and size that were averaged in the same participants across two visits to account for the cumulative exposure to these parameters [29,30]. Our study is limited by the use of the MoCA as the only tool for assessment of cognitive function, and lack of CSF collection for participants in the present study. Therefore, future studies are needed to characterize association between HDL structure/function with more comprehensive neurocognitive assessment in all domains. However, the use of CSF samples in populations with normal cognition as a screening tool is impractical. Nevertheless, changes in brain structure in cognitively intact adults, such as reduced GMV as a process of normal aging, are thought to be related to downstream changes in cognitive function later in life [46]. Therefore, having higher GMV in mid-life may aid in preventing cognitive decline associated with brain atrophy as part of normal aging in late life.

## 5. Conclusions

Our data suggest that higher levels of HDL cholesterol efflux capacity and higher levels of small circulating HDL particles are positively associated with greater grey matter volume independent of traditional risk factors such as age, smoking status, time spent in daily physical activity, and education level. Future studies should focus on determining if adopting strategies to maintain optimal HDL functionality and HDL composition during midlife results in the preservation of gray matter volume into late life.

## Figures and Tables

**Table 1:** Participant characteristics in each phase of the Dallas Heart Study.

Variable	DHS-1	DHS-2	Average (DHS-1 and DHS-2)
Female (%)		1060 (58%)	
Black participants		863 (47%)	
Age (years old)	43.7 ± 9.8	51.0 ± 9.7	
BMI (kg/m^2^)	30.5 ± 7.4	31.2 ± 7.4	30.9 ± 7.2
Fasting plasma glucose	100.2 ± 36.0	102.0 ± 34.0	101.1 ± 30.8
eGFR (mL/min per 1.73 m^2^)	99.2 ± 22.7	93.1 ± 26.5	96.2 ± 22.7
Systolic blood pressure (mm Hg)	123 ± 18	132 ± 19	128 ± 16
HDL particle, μmol/L	20.9 ± 3.9	22.7 ± 3.6	21.8 ± 3.2
Small HDL particle, μmol/L	15.0 ± 3.5	15.0 ± 3.6	15.0 ± 3.0
Medium HDL particle, μmol/L	3.7 ± 2.8	5.6 ± 2.6	4.7 ± 2.4
Large HDL particle, μmol/L	2.2 ± 1.6	2.1 ± 1.5	2.2 ± 1.4
HDL cholesterol, mg/dL	51.1 ± 14.7	55.7 ± 13.2	53.4 ± 12.8
Apolipoprotein A-I, mg/dL	127.6 ± 29.6	142.9 ± 26.4	135.3 ± 25.3
HDL cholesterol efflux capacity (AU)	1.03 ± 0.32	0.83 ± 0.24	0.93 ± 0.21
Current smoker (%)	440 (24.1%)	384 (21.3%)	
MVPA (min/day)		39 (95% CI 37.2–40.7)	
ApoE-ε4 carrier (%)		566 (31.5%)	
Education Status			
Less than high school		257 (14%)	
High school		434 (24%)	
College level		957 (53%)	
Above college		172 (10%)	

Data are presented as the mean ± SD, mean with 95% SD, or frequency with percentage. BMI: body mass index; eGFR: estimated glomerular filtration rate; HDL: high-density lipoprotein; MVPA: moderate-to-vigorous physical activity.

**Table 2:** Multivariable associations between high-density lipoprotein measures and grey matter volume, white matter hyperintensities, and total Montreal Cognitive Assessment score.

Outcome: Log GMV/TCV for Whole Cohort (*n* = 1010)			
Variable	Β ^a^	95% CI	*p*-value
Total HDL-P	0.011	−0.039, 0.060	0.668
Small HDL-P	0.063	0.014, 0.111	0.012 ^b^
Medium HDL-P	−0.045	−0.095, 0.004	0.072
Large HDL-P	−0.041	−0.094, 0.012	0.130
HDL-C	−0.040	−0.092, 0.014	0.149
ApoAI	−0.020	−0.070, 0.035	0.516
HDL-CEC *	0.078	0.029, 0.126	0.002 ^b^
Outcome: Log WMH/TCV for Whole Cohort (*n* = 1010)			
HDL-P	0.003	−0.055, 0.061	0.920
Small HDL-P	−0.014	−0.072, 0.043	0.623
Medium HDL-P	0.0001	−0.058, 0.058	0.997
Large HDL-P	0.042	−0.020, 0.105	0.181
HDL-C	0.018	−0.045, 0.081	0.573
ApoAI	0.012	−0.050, 0.073	0.713
HDL-CEC *	0.033	−0.024, 0.091	0.256
Outcome: MoCA for Whole Cohort (*n* = 1176)			
HDL-P	0.021	−0.028, 0.070	0.408
Small HDL-P	−0.005	−0.054, 0.044	0.844
Medium HDL-P	0.017	−0.032, 0.067	0.498
Large HDL-P	0.033	−0.020, 0.086	0.218
HDL-C	0.044	−0.009, 0.097	0.103
ApoAI	0.036	−0.016, 0.088	0.171
HDL-CEC *	−0.009	−0.059, 0.042	0.737

^a^ β (standardized regression coefficients) and 95% confidence intervals. ^b^ Indicates statistical significance. All exposures are an average from DHS 1 and DHS 2. Model: Multivariable models were performed with a single HDL parameter and adjusted for age, sex, race/ethnicity, smoking status, education level, and MVPA from DHS-2; and BMI, systolic BP, fasting plasma glucose, and eGFR averaged from DHS-1 and DHS-2. * Includes HDL-C as covariate in the model plus the above adjustments. Abbreviations: ApoAI: apolipoprotein AI; BMI: body mass index; BP: blood pressure; CEC: cholesterol efflux capacity; DHS: Dallas Heart Study; eGFR: estimated glomerular filtration rate; GMV: grey matter volume; HDL: high-density lipoprotein; MVPA: moderate-to-vigorous physical activity; TCV: total cranial volume; WMH: white matter hyperintensities.

## Data Availability

The datasets generated and analyzed during the current study are available from the corresponding author upon reasonable request.
